# Minimally invasive sacroiliac joint fusion: one-year outcomes in 18 patients

**DOI:** 10.1186/1750-1164-7-12

**Published:** 2013-09-16

**Authors:** John Cummings, Robyn A Capobianco

**Affiliations:** 1 Community Neurosurgical Care, 1400 N. Ritter Ave, Suite 479, Indianapolis, IN 46219, USA; 2 SI-BONE Inc., 3055 Olin Ave. Suite 2200, San Jose, CA 95128, USA

**Keywords:** Minimally invasive surgery, Sacroiliac joint, Arthrodesis, Previous spine surgery

## Abstract

**Background:**

Sacroiliac joint (SI) pain is an often-overlooked cause of low back pain due, in part, to lack of specific findings on radiographs and symptoms mimicking other back-related disorders. We report our experience with minimally invasive (MIS) SI joint arthrodesis using a series of triangular, titanium plasma spray (TPS) coated implants in patients refractory to conservative care.

**Methods:**

We report outcomes from 18 patients with 12 months of postoperative follow-up.

Demographics, complications, and clinical outcomes using visual analog scale (VAS) for pain, Oswestry Disability Index (ODI) for back function and SF-12 for quality of life were collected preoperatively and at 3, 6 and 12 months post-operatively.

**Results:**

Mean age was 64 years and 67% of patients were female. There were no intraoperative complications and one explant at three months for malposition.

All patient-reported outcomes showed both clinically and statistically significant improvement at 12 months (p < 0.001 for each of the following): VAS improved by 6.6 points, ODI scores improved by −37.5 points. One year SF-12 physical and mental component (PCS, MCS) scores approximated population normal scores for both physical and mental functioning. Patient satisfaction with outcomes was high at 95%; 89% said would have the same surgery again.

**Conclusions:**

MIS SI joint fusion using a series of triangular porous TPS coated titanium implants is a safe and effective procedure for patients with SI joint disorders who have failed conservative care.

## Background

Sacroiliac (SI) joint pain is an often-overlooked cause of low back pain. This may be due in part to the lack of visible abnormalities on plain radiographs and the fact that SI joint pain may mimic hip, lumbar radicular disorders or pain of discogenic origin [1,2]. Patients with SI joint problems may present with low back, groin, gluteal, and/or radicular pain leading to the potential for inaccurate diagnosis and treatment [3-5].

Historically, though the sacroiliac joint was a prominent initial focus of attention in the early 1900s as a significant generator of low back pain (LBP), as more reliably diagnosed conditions such as herniated discs and facet arthropathy became better understood, less focus was placed on the SI joint [6]. A large study conducted by Bernard and Kirkaldy-Willis found that 22.5% of patients diagnosed with nonspecific LBP actually had SI joint problems [1]. A more recent study reported a prevalence rate between 15-30% of patients presenting with low back pain [2]. Several studies investigating the affect of lumbar fusion on SI joint disorders have shown an SI joint pain prevalence rate of up to 61% after lumbosacral fusion and significant radiographic SI joint degeneration in up to 75% of post-lumbar fusion patients at 5 years [7-9].

**Figure 1:**
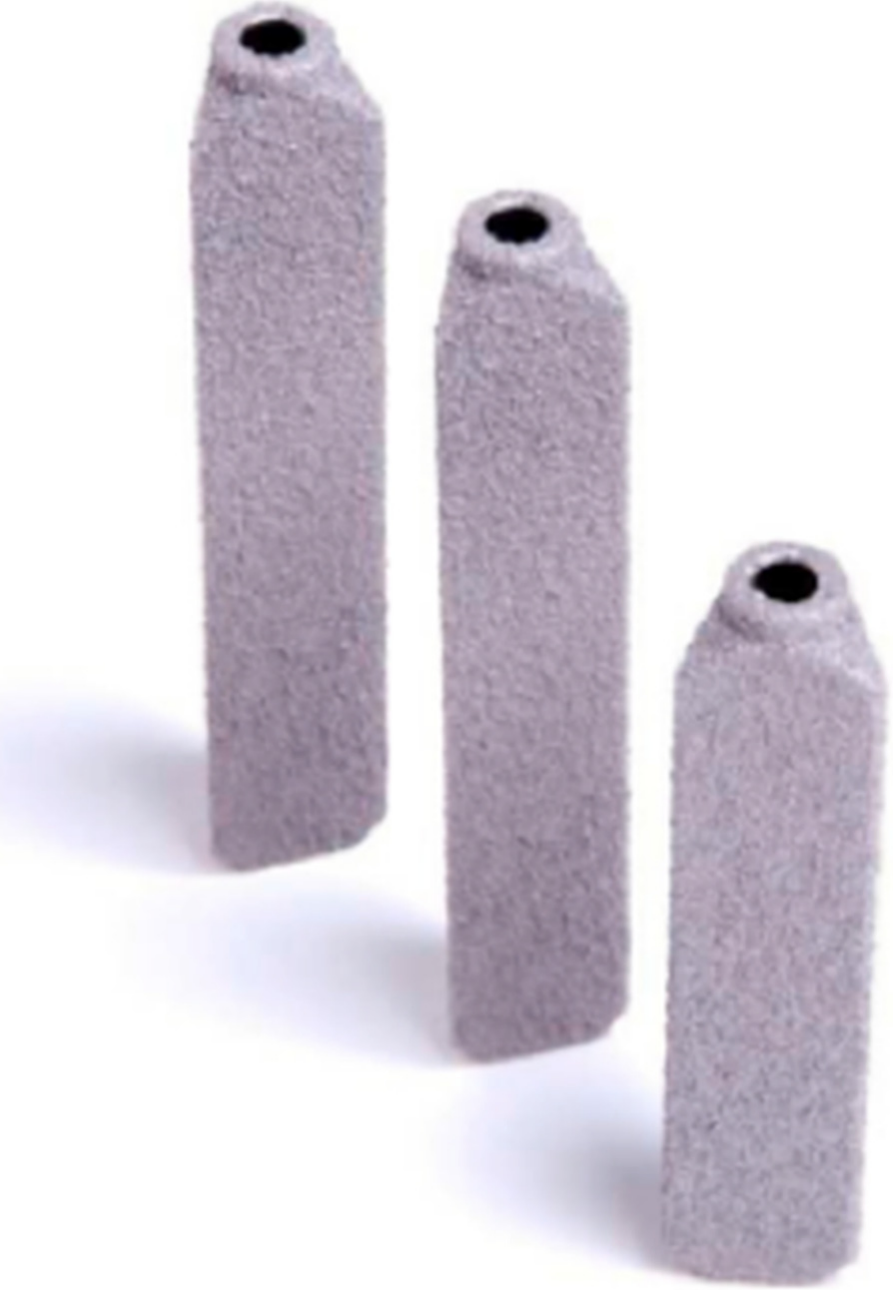
Triangular porous plasma coated implants (iFuse® Implant System, SI-BONE Inc, San Jose, CA).

Open arthrodesis of the SI joint was commonly performed in the early 1900s. However, open SI joint fusion is less common now as it requires relatively large incisions, significant bone harvesting, and lengthy hospital stays; moreover, patients must avoid weight -bearing for several months postoperatively [10-13]. Minimally invasive SI joint fusion has become available with various devices. Recent reports of a minimally invasive arthrodesis system have shown promising outcomes [14,15]. The surgical procedure involves the placement of three triangular implants across the SI joint (Figure 1). Second site bone harvesting or graft is not required as the porous titanium plasma spray (TPS) coating on the implants results in biological fixation of the implant in bone. Herein we report safety and effectiveness outcomes of patients undergoing MIS SI joint fusion with a series of triangular titanium, porous TPS coated implants (iFuse Implant System, SI-BONE, Inc., San Jose, CA) in a single surgeon’s private practice.

## Methods

A medical chart review was undertaken to identify patients who underwent MIS SI joint fusion surgery at a community based spine practice more than 12 months ago. Patients were excluded if concomitant spine procedures were performed and if no preoperative or follow up outcomes were available. A total of 34 patients were identified: 24 had one year follow up, 18 underwent unilateral surgery and are included in our analysis. All patients were treated between September 2011 and April 2012. Data collected included patient demographics, medical history, and complications of surgery. Clinical outcomes were collected prospectively preoperatively and at 6 weeks, 3 months, 6 and 12 months postoperatively. Institutional Review Board Approval (Community Health Network) was obtained before beginning this study.

### Diagnosis

Using a combination of detailed history, clinical exam, imaging and positive diagnostic injections, all patients were diagnosed with either degenerative sacroiliitis or sacroiliac joint disruption. A thorough physical and clinical exam was performed in order to establish the pain generator as accurately as possible in this complex population. A positive result on 3 or more pain provocation tests such as Gaenselen’s, flexion abduction external rotation (FABER), compression, distraction and thigh thrust, was used as criteria for further testing to confirm the SI joint as the pain generator [16-19]. Diagnostic imaging studies such as x-ray, CT and MRI, while not sensitive in diagnosing disorders of the SI joint, are helpful in ruling out pathology in the lumbar spine and hip [17]. When clinical, physical and imaging findings were congruent, patients were sent for image-guided diagnostic injections of the SI joint. A positive result was defined as a 75% reduction in pain immediately following injection of local anesthetic [20]. Conservative treatment consisting of medication optimization, physical therapy and SI joint injections was prescribed for a course of at least 6 months before offering the patient surgery.

### Technique overview

**Figure 2:**
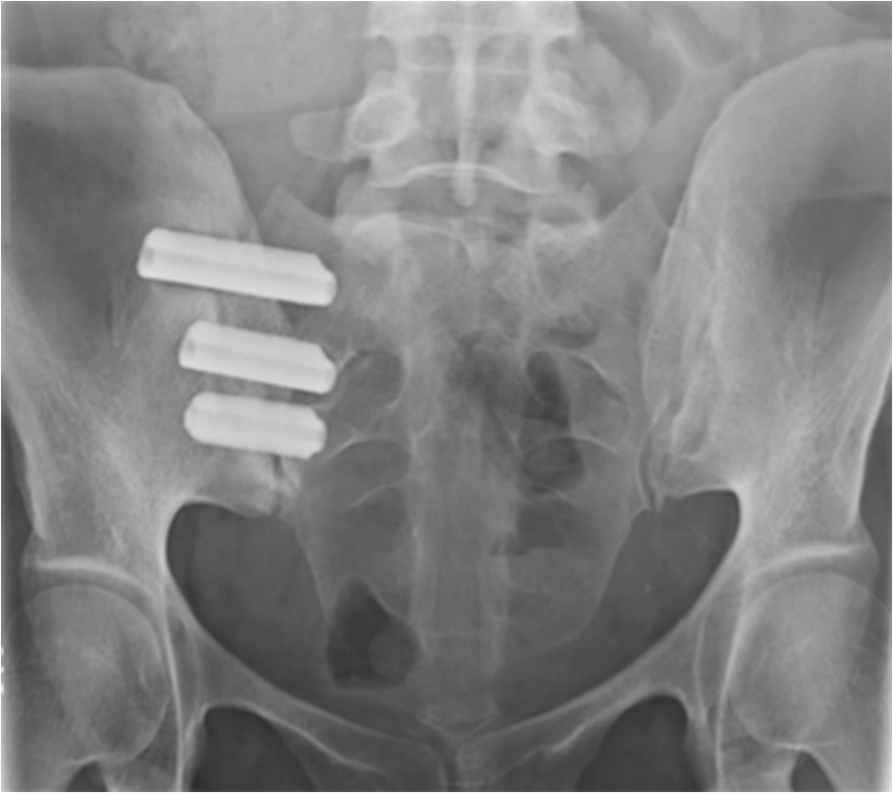
Postoperative radiograph demonstrating placement of three implants across the SI joint.

Minimally invasive SI joint fusion surgery was performed in all cases by a single neurosurgeon in private practice. This procedure involves fixation of the SI joint by placing a series (typically three) of triangular, titanium porous TPS coated implants across the SI joint. No second site bone harvesting is required due to the biological fixation properties of the porous TPS coating. The patient is placed in the prone position on a radiolucent table to facilitate the use of intraoperative fluoroscopy. After general endotracheal anesthesia is administered, the patient is prepped in the normal sterile fashion. A 3 cm lateral incision is made into the buttock region and the gluteal fascia is bluntly dissected to reach the outer table of the ilium. A Steinmann pin is passed through the ilium across the SI joint to the center of the sacrum (lateral to the neural foramen). After a soft tissue protector is passed over the pin, a hand drill is used to create a pathway and decorticate the bone. Finally, a triangular broach is used to further decorticate the bone and prepare the pathway to receive the first implant. Using a pin guidance system, a total of three implants are placed. The most cephalad implant is seated within the sacral ala. The second implant is generally located above or adjacent to the S1 foramen and the third between the S1 and S2 foramen (Figure 2). The incision is then irrigated and the tissue layers are sequentially closed. Patients begin physical therapy on day 1 postop and are instructed to ambulate with the assistance of a walker or crutch for the first 3 weeks. During the 3–6 week postoperative period, patients progress to full weight bearing with a regimen of flexibility and strengthening exercises. After 6 weeks of gradual return to full weight bearing, patients are back to unrestricted activity and are encouraged to continue core stabilization exercises as well as weight and cardiovascular training.

### Clinical outcome assessments

Patient reported clinical outcomes were collected prospectively prior to surgery to establish baseline values and at 6 weeks, 3, 6 and 12 months post-operatively. The following instruments were used: visual analog scale (VAS) for pain, Oswestry Disability Index (ODI) (version 2.1) for back related function, and Short Form 12 (SF-12) for quality of life [21,22]. Satisfaction at 12 months was assessed by asking the patient 2 questions: “describe your satisfaction with this surgical outcome” and “would you elect the have the same surgery again?” Possible responses were “very, somewhat, or not really” for question 1 and “definitely, likely, or definitely not” for question 2.

### Statistical analysis

Baseline demographic variables were summarized with mean, standard deviation and frequency tables. Changes in clinical outcomes variables (VAS, ODI, SF-12 PCS and MCS) were evaluated using a paired *t*-test. Analysis of variance (ANOVA) was used to evaluate differences in outcomes across subgroups. Subgroup variables analyzed were history of prior lumbar fusion, hypertension, body mass index (BMI) by category, age (> or < 65 years) and sex. BMI categories were defined using World Health Organization (WHO) standards: <25 normal, 25–30 overweight, >30 obese. All analysis was performed using SAS (version 9.0, Cary, NC).

Clinical improvement was defined using minimum clinically important difference (MCID) and substantial clinical benefit (SCB) values available in the literature. Currently there are no reported MCID or SCB values for SI joint fusion. We chose MCID thresholds for VAS pain and ODI improvement reported in the Spine Patient Outcomes Research Trial (SPORT): ≥2 points for VAS pain and ≥12.8 for ODI [23]. MCID values for SF-12 PCS and MCS (8.8 and 9.3 points respectively) were acquired from a study on lumbar spine surgery for adjacent segment disease, since the majority of the patients in our cohort had a history of previous lumbar spine fusion [24]. Substantial clinical benefit (SCB) values were chosen using criteria by Glassman et al. for patients undergoing lumbar spine arthrodesis [25]. SCB for ODI is defined as an 18.8-point improvement or final score of <31.3 points. SCB for SF-36 PCS is defined as 6.2-point improvement or final score of ≥ 35.1 and SCB for pain is 2.5-point decrease or raw score of < 3.5.

ODI scores may be described in terms of degree of disability. A score of 0-20% represents minimal disability, 21-40% moderate disability, 41-60% severe disability and 61-80% crippled (Table 1). Scores greater than 81% were not seen in our population and are described as “either bed bound or exaggerating their symptoms.”

**Table 1:** **Score interpretation of the Oswestry disability questionnaire**[21]**for low back pain**

**0% to 20%: minimal disability:**	The patient can cope with most living activities. Usually no treatment is indicated apart from advice on lifting sitting and exercise.
**21%-40%: moderate disability:**	The patient experiences more pain and difficulty with sitting, lifting and standing. Travel and social life are more difficult and they may be disabled from work. Personal care, sexual activity and sleeping are not grossly affected and the patient can usually be managed by conservative means.
**41%-60%: severe disability:**	Pain remains the main problem in this group but activities of daily living are affected. These patients require a detailed investigation.
**61%-80%: crippled:**	Back pain impinges on all aspects of the patient’s life. Positive intervention is required.
**81%-100%:**	These patients are either bed-bound or exaggerating their symptoms.

## Results

Eighteen patients underwent unilateral MIS SI joint fusion and had 1-year follow-up. No patient had a concomitant procedure. No intraoperative complications were observed and blood loss was minimal (<50 cc) in all cases. Mean age was 64 years (range 39–81) (Table 2). Patients were primarily white (83%) and female (67%). The large majority (89%) of patients in this cohort were obese (BMI > 30) or overweight (BMI 25–30), and 44% were hypertensive. Most patients (83%) had a history of previous lumbar spine surgery that included: fusion at one or more levels (73%), decompression (13%), and discectomy (13%).

**Table 2:** Patient demographics

**N**	**18**
Age	64 (range 39–81) (12.2 SD)
Sex	67% (12) F, 33% (6) M
Race	83% (15) Caucasian
	17% (3) African American
BMI mean	31
Obese (>30)	61% (11)
Overweight (25–30)	28% (5)
Normal (<25)	11% (2)
Diabetes	21% (4)
Smoking status	2 current, 5 former
Hypertension	44% (8)
Prior lumbar spine surgery	83% (15):
	2 microdiscectomy
	11 fusion
	2 decompression
Side treated	11 L (61%), 7R (39%)

VAS pain scores improved clinically and statistically at all postoperative time points (Table 3). Mean (SD) scores improved from 8.9 (± 1.9) at baseline to 2.3 (2.1) at 12 months with a mean change of −6.56 points (p < .0001) for this time period (Table 4). A clinically significant benefit, defined as ≥2 point change from baseline, was observed in 90% of patients [23].

**Table 3:** Clinical outcomes

	**Baseline**		**6wk**		**3mo**		**6mo**		**12mo**	
	**n**	**Mean (SD)**	**n**	**Mean (SD)**	**n**	**Mean (SD)**	**n**	**Mean (SD)**	**n**	**Mean (SD)**
VAS	18	8.9 (1.9)	12	3.1 (1.6)	13	3.0 (2.5)	10	4.4 (3.2)	18	2.3 (2.1)
ODI	18	52.7 (18.8)	5	22.8 (19.8)	10	16.8 (16.4)	3	29.0 (12.7)	18	13.2 (12.6)
SF-12 PCS	18	32.3 (6.4)	6	38.1 (10.1)	9	44.8 (10.2)	3	37.4 (1.9)	18	44.6 (10.5)
SF-12 MCS	18	37.6 (10.2)	6	42.6 (13.1)	9	51.2 (11.3)	3	36.1 (2.1)	18	53.8 (9.5)

**Table 4:** 12 month clinical outcomes

	**Baseline**	**12 mo**	**Change**	**p**
VAS	8.9 (1.9)	2.3 (2.1)	−6.56	<0.001
ODI	52.6 (18.8)	13.2 (12.6)	−37.54	<0.001
SF-12 PCS	32.3 (6.4)	44.6 (10.5)	11.19	<0.005
SF-12 MCS	37.8 (10.4)	53.8 (9.5)	20.37	<0.001

Back-related function measured on ODI improved clinically and statistically. Improvement was observed as early as the 6-week post-operative visit. Mean (SD) scores decreased from 52.7 (18.8) at baseline to 13.2 (12.6) at 12 months with a mean change of −37.5 points (p < .0001). Baseline ODI scores categorized 61% of patients as “severely disabled,” 17% as “crippled,” and 2% each as “moderately” and “minimally disabled.” At the 12 month visit, 89% of patients were classified into the “minimal disability category” and the remaining 2 patients scored into the “moderate disability” category [21]. Both of these patients were considered as “severely disabled” at baseline with multiple back pain complaints. Substantial clinical benefit was achieved for 89% of patients.

Quality of life as measured on SF-12 using the aggregate physical and mental component summary (PCS and MCS) scores showed a clinically and statistically significant improvement (p < 0.005, p < 0.001 respectively). Mean PCS score rose from 32.3 (6.4) at baseline to 44.6 (10.5) at 12 months, representing an improvement in physical health by 11.2 points. MCS scores improved from 37.8 at baseline to 53.8 at 12 months, a mean improvement of 20.4 points. The SF-12 outcome measure scale of 0–100 was designed such that a mean score of 50 with a standard deviation of 10 represents average health status (United States population). The improvement gained in our patient population is commensurate with reported age category scores of the general US population suggesting a return of health to near normal levels. SCB and MCID were reached for 72% of patients on SF-12 PCS. MCID (no SCB was available) for SF-12 MCS was reached in 78% of patients.

More than half of patients were “very satisfied” with the surgical outcome only 1 patient (5.6%) was “not really” satisfied (Table 5). When asked if they would have the same surgery for the same result, 83% responded “definitely,” 5.6% answered “likely,” and 11% indicated “definitely not.”

**Table 5:** Satisfaction

**Satisfaction with**		**Would you have the same**	
**surgical outcomes**		**surgery for the same result?**	
Very	10 (55.6%)	Definitely	15 (83.3%)
Somewhat	7 (38.9%)	Likely	1 (5.6%)
Not really	1 (5.6%)	Definitely not	2 (11.1%)

Most (84%) patients had undergone previous lumbar spine surgery. A subgroup analysis of improvement in pain amongst those with and without a history of prior lumbar spine fusion showed no difference in clinical outcomes. Analysis of variance showed that other variables (history of hypertension, diabetes, smoking, age and gender) had no affect on pain (VAS), function (ODI), or quality of life (SF-12) improvements. Of interest, 89% of patients had a BMI of greater than 25 indicating they were overweight or obese. Although only a small percentage of patients fell within the “normal” BMI range, subgroup analysis revealed no statistically significant effect of BMI on outcomes.

### Complications

There were no intraoperative complications. Postoperatively, one patient had fluid retention, three patients experienced transient trochanteric bursitis treated with medical management and one patient had transient toe numbness of unclear relationship to SI joint fusion surgery (Table 6). One operative site hematoma occurred. In this same patient, a selective nerve root block was performed at S1 to alleviate pain from a resulting piriformis spasm. One patient experienced new radicular pain at the 3 month visit. CT scan revealed the most cephalad implant was seated just outside the anterior cortex, resulting in an irritation of the L5 nerve root. The implant was subsequently removed and the patient had a complete resolution of radicular symptoms. The resulting major complication rate as defined by Lebude et al. [26] for this cohort was 5%.

**Table 6:** Complications

**Complication**	**n**
Trochanteric bursitis	3
Hematoma	1
Fluid retention	1
Toe numbness	1
Implant malposition	1

## Discussion

SI joint symptoms can present as pain in the SI joint, low back, hip, groin, or buttock, and abnormalities are rarely seen on plain radiographs alone [1,17]. To accurately diagnose the SI joint as the pain generator in patients with low back pain symptoms requires a combination of detailed clinical history, physical exam maneuvers that stress the SI joint, and marked pain relief on diagnostic SI joint block [8,27].

Recent reports of MIS approaches to SI joint arthrodesis using hollow modular anchorage (HMA) screws packed with demineralized bone matrix show relatively good clinical results, but with room for improvement [28-30]. However, recent reports suggest that this technique may not be appropriate for patients with a history of instrumented spinal surgery. Mason et al. reported significantly worse outcomes after SI joint fusion using HMA screws in patients with a history of previous lumbar spine surgery [30]. In contrast, a recently published report of outcomes after SI joint fusion using the triangular implants reported herein (iFuse Implant System) showed clinically and statistically significant improvements in pain and function independent of a prior history of lumbar spine fusion [14]. Similarly, there was no difference in outcomes in the current study between patients with and without history of lumbar spinal fusion. Recent case series reports using the same technique described herein report favorable results with minimal complications and no suggestion of implant loosening [14,15,31,32].

Advantages of a MIS approach over standard open fusion include a small incision, minimal blood loss, bone and ligament preservation, and a relatively short period of immobilization. The triangular shape combined with an interference fit of the titanium implant used in this cohort was designed to minimize rotation and micromotion in order to avoid the issues of loosening, backing out and implant protrusion that can be encountered with traditional screws [33]. Due to both the porous nature of the implant’s titanium plasma spray coating along with the implant versus cage design, iliac crest bone harvesting is not required.

Post-operative complications were minimal and the most common complaint was transient trochanteric bursitis. This is neither uncommon nor unexpected, and can be a result of altered gait pattern due to low back or hip pain, post-operative hip abductor weakness, increased activity levels and other trauma in the region [34]. One patient presented with pain in the L5 distribution 3 months after device placement. Impingement of the distal end of the most cephalad implant on the L5 nerve root was revealed on CT scan. After the implant was removed, the patient’s pain resolved completely. This patient had an otherwise excellent outcome with a 12-month pain score of 1, an ODI score of 6, and normal SF-12 PCS and MCS scores. This finding underscores the importance of accurate device placement during surgery.

Most patients in this cohort had a history of previous lumbar spine surgery with the most common procedure being instrumented fusion (75%). It is unclear whether the degradation of the SI joint was a result of adjacent segment disease (ASD) or de novo degeneration. Using pre-determined thresholds (MCID or SCB where available) for clinically significant improvement, the success rates observed in our study were high: 90% of patients met this threshold for improvement in pain (VAS), 89% for back related function (ODI), 72% for physical quality of life (SF-12 PCS), 78% for mental quality of life (SF-12 MCS). Moreover, the presence of prior lumbar spine fusion did not seem to affect improvement of pain and functional scores.

Although the current study sample size is small, the results are very encouraging. Favorable outcomes in this cohort underscore the necessity to suspect the SI joint as a pain generator in patients with low back pain especially after lumbar spine surgery. Results for this reported procedure in patients with instrumented fusion are as favorable as in patients with no prior lumbar surgical history. This procedure has the potential to significantly benefit the elderly population, who are not candidates for other conventional techniques due to poor bone quality, delayed healing and reduced mobility. Half of the patients in this cohort are 65 years or older and a sub group analysis revealed no difference in outcomes at one year for patients < or > 65 years. This segment of the population is not likely to respond well to physical therapy alone in part because of the degenerative nature of SI joint disease. The MIS procedure described herein affords this segment of the population an opportunity to regain mobility, alleviate SI joint and low back pain caused by SI joint issues and experience an improved quality of life.

## Conclusion

Minimally invasive SI joint fusion using a series of triangular porous TPS coated implants is effective in improving pain, function and quality of life in patients with disorders of the SI joint who have failed conservative treatment. The complication rate was low.

## Competing interests

JC is a paid consultant for SI-BONE, Inc. RC is an employee of SI-BONE, Inc.

## Authors’ contributions

JC performed all surgeries, patient assessments, data collection, critical revision of the manuscript and gave final approval of the manuscript. RC performed data analysis, interpretation, and drafted the manuscript. Both authors read and approved the final manuscript.

## Authors’ information

JC is a board certified neurosurgeon specializing in minimally invasive treatment of spinal disorders. RC is a clinical research professional and medical writer.

## References

[B1] Bernard TN Kirkaldy-Willis WH Recognizing specific characteristics of nonspecific low back pain Clin Orthop Relat Res 1987 217 266 280 2951048

[B2] Sembrano JN Polly DW How often is low back pain not coming from the back? Spine 2009 34 1 E27 E32 10.1097/BRS.0b013e31818b8882 19127145

[B3] Foley BS Buschbacher RM Sacroiliac joint pain: anatomy, biomechanics, diagnosis, and treatment Am J Phys Med Rehabil 2006 85 12 997 1006 10.1097/01.phm.0000247633.68694.c1 17117004

[B4] Schwarzer AC Aprill CN Bogduk N The sacroiliac joint in chronic low back pain Spine 1995 20 1 31 37 10.1097/00007632-199501000-00007 7709277

[B5] Weksler N Velan GJ Semionov M Gurevitch B Klein M Rozentsveig V The role of sacroiliac joint dysfunction in the genesis of low back pain: the obvious is not always right Arch Orthop Trauma Surg 2007 127 10 885 888 10.1007/s00402-007-0420-x 17828413

[B6] Wise CL Dall BE Minimally invasive sacroiliac arthrodesis: outcomes of a new technique J Spinal Disord Tech 2008 21 8 579 584 10.1097/BSD.0b013e31815ecc4b 19057252

[B7] DePalma MJ Ketchum JM Saullo TR Etiology of chronic low back pain in patients having undergone lumbar fusion Pain Med 2011 12 5 732 739 10.1111/j.1526-4637.2011.01098.x 21481166

[B8] Liliang P-C Lu K Liang C-L Tsai Y-D Wang K-W Chen H-J Sacroiliac joint pain after lumbar and lumbosacral fusion: findings using dual sacroiliac joint blocks Pain Med 2011 12 4 565 570 10.1111/j.1526-4637.2011.01087.x 21463470

[B9] Ha K-Y Lee J-S Kim K-W Degeneration of sacroiliac joint after instrumented lumbar or lumbosacral fusion: a prospective cohort study over five-year follow-up Spine 2008 33 11 1192 1198 10.1097/BRS.0b013e318170fd35 18469692

[B10] Buchowski JM Kebaish KM Sinkov V Cohen DB Sieber AN Kostuik JP Functional and radiographic outcome of sacroiliac arthrodesis for the disorders of the sacroiliac joint Spine J 2005 5 5 520 528 discussion 529 10.1016/j.spinee.2005.02.022 16153580

[B11] Giannikas KA Khan AM Karski MT Maxwell HA Sacroiliac joint fusion for chronic pain: a simple technique avoiding the use of metalwork Eur Spine J 2004 13 3 253 256 10.1007/s00586-003-0620-1 14648303 PMC3468135

[B12] Smith-Petersen MN Arthrodesis of the sacroiliac joint. A new method of approach J Bone Joint Surg Am 1921 3 8 400 405

[B13] Moore MR Surgical treatment of chronic painful sacroiliac joint dysfunction. Movement, stability, and low back pain : the essential role of the pelvis 1997 New York: Churchill Livingstone 563 572

[B14] Rudolf L MIS fusion of the SI joint: does prior lumbar spinal fusion affect patient outcomes? Open Orthop J 2013 7 163 168 10.2174/1874325001307010163 23730380 PMC3664440

[B15] Sachs D Capobianco R One year successful outcomes for novel sacroiliac joint arthrodesis system Ann Surg Innovat Res 2012 6 1 13 16 10.1186/1750-1164-6-13 PMC3561253 23270468

[B16] Szadek KM van der Wurff P van Tulder MW Zuurmond WW Perez RSGM Diagnostic validity of criteria for sacroiliac joint pain: a systematic review J Pain 2009 10 4 354 368 10.1016/j.jpain.2008.09.014 19101212

[B17] Simopoulos TT Manchikanti L Singh V Gupta S Hameed H Diwan S A systematic evaluation of prevalence and diagnostic accuracy of sacroiliac joint interventions Pain Physician 2012 15 3 E305 E344 22622915

[B18] Gaenslen FJ Sacro-iliac arthrodesis: indications, author’s technic and end-results JAMA 1927 89 24 2031 2035 10.1001/jama.1927.02690240023008

[B19] Robinson HS Brox JI Robinson R Bjelland E Solem S Telje T The reliability of selected motion- and pain provocation tests for the sacroiliac joint Man Ther 2007 12 1 72 79 10.1016/j.math.2005.09.004 16843031

[B20] Maigne JY Aivaliklis A Pfefer F Results of sacroiliac joint double block and value of sacroiliac pain provocation tests in 54 patients with low back pain Spine 1996 21 16 1889 1892 10.1097/00007632-199608150-00012 8875721

[B21] Fairbank JC Pynsent PB The Oswestry disability index Spine 2000 25 22 2940 2952 10.1097/00007632-200011150-00017 11074683

[B22] Ware J Kosinski M Keller S A 12-item short-form health survey: construction of scales and preliminary tests of reliability and validity Med Care 1996 34 3 220 233 10.1097/00005650-199603000-00003 8628042

[B23] Copay AG Glassman SD Subach BR Berven S Schuler TC Carreon LY Minimum clinically important difference in lumbar spine surgery patients: a choice of methods using the Oswestry disability index, medical outcomes study questionnaire short form 36, and pain scales Spine J 2008 8 6 968 974 10.1016/j.spinee.2007.11.006 18201937

[B24] Parker SL Mendenhall SK Shau D Adogwa O Cheng JS Anderson WN Determination of minimum clinically important difference in pain, disability, and quality of life after extension of fusion for adjacent-segment disease J Neurosurg Spine 2012 16 1 61 67 10.3171/2011.8.SPINE1194 21962034

[B25] Glassman SD Copay AG Berven SH Polly DW Subach BR Carreon LY Defining substantial clinical benefit following lumbar spine arthrodesis J Bone Joint Surg 2008 90 9 1839 1847 10.2106/JBJS.G.01095 18762642

[B26] Lebude B Yadla S Albert T Anderson DG Harrop JS Hilibrand A Defining “Complications” in spine surgery J Spinal Disord Tech 2010 23 8 493 500 10.1097/BSD.0b013e3181c11f89 20124913

[B27] Van der Wurff P Buijs EJ Groen GJ A multitest regimen of pain provocation tests as an aid to reduce unnecessary minimally invasive sacroiliac joint procedures Arch Phys Med Rehabil 2006 87 1 10 14 10.1016/j.apmr.2005.09.023 16401431

[B28] Al-Khayer A Hegarty J Hahn D Grevitt MP Percutaneous sacroiliac joint arthrodesis: a novel technique J Spinal Disord Tech 2008 21 5 359 363 10.1097/BSD.0b013e318145ab96 18600147

[B29] Khurana A Guha AR Mohanty K Ahuja S Percutaneous fusion of the sacroiliac joint with hollow modular anchorage screws: clinical and radiological outcome J Bone Joint Surg Br 2009 91 5 627 631 19407297 10.1302/0301-620X.91B5.21519

[B30] Mason LW Chopra I Mohanty K The percutaneous stabilisation of the sacroiliac joint with hollow modular anchorage screws: a prospective outcome study Eur Spine J 2013 epub ahead of print 10.1007/s00586-013-2825-2 PMC3804714 23686478

[B31] Miller L Reckling WC Block JE Analysis of postmarket complaints database for the iFuse SI Joint Fusion System: a minimally invasive treatment for degenerative sacroiliitis and sacroiliac joint disruption Med Dev Evid Res 2013 6 77 84 10.2147/MDER.S44690 PMC3673964 23761982

[B32] Kim JT Rudolf LM Glaser JA Outcome of percutaneous sacroiliac joint fixation with porous plasma-coated triangular titanium implants: an independent review Open Orthop J 2013 7 51 56 10.2174/1874325001307010051 23525073 PMC3601344

[B33] Routt MLC Simonian PT Inaba J Iliosacral screw complications Operat Tech Orthop 1997 7 7 206 220

[B34] Shbeeb M Matteson E Trochanteric bursitis (greater trochanteric pain syndrome) Mayo Clinic Proc 1996 71 565 569 10.4065/71.6.565 8642885

